# Predictors of Postoperative Hypocalcemia and Hypoparathyroidism Following Thyroidectomy in Hanoi, Vietnam

**DOI:** 10.5812/ijem-146358

**Published:** 2024-06-26

**Authors:** Khanh Nam Do, Phuong Thi Duong, Toi Lam Phung, Yen Thi Duong, Giang Truong Hoang, Huong Thi Le

**Affiliations:** 1 Department of Nutrition and Food Safety, School of Preventive Medicine and Public Health, Hanoi Medical University, Hanoi, Vietnam; 2 Department of Nutrition and Dietetics, Hanoi Medical University Hospital, Hanoi Medical University, Hanoi, Vietnam; 3 Health Strategy and Policy Institute, Ministry of Health, Hanoi, Vietnam; 4 Department of Clinical Nutrition, Vietnam National Cancer Hospital, Hanoi, Vietnam

**Keywords:** Hypocalcemia, Thyroidectomy, Hypoparathyroidism, Risk Factors

## Abstract

**Background:**

Hypocalcemia is the most frequent complication of thyroid surgeries. Hypocalcemia is the most common complication following thyroid surgeries and is crucial in managing patients with thyroid cancer.

**Objectives:**

This study aimed to describe hypocalcemia after thyroidectomy and evaluate the factors associated with postoperative hypocalcemia.

**Methods:**

A cross-sectional study was conducted on 91 patients with thyroid cancer at Hanoi Medical University Hospital. Hypocalcemia was defined as serum calcium levels lower than 2.1 mmol/L, measured 24 hours after surgery.

**Results:**

In the postoperative period, 27.5% of the patients exhibited hypocalcemia, with distinct prevalence rates observed between the total thyroidectomy group (47.6%) and the thyroid lobectomy group (10.2%). Concurrently, hypoparathyroidism manifested in 15.4% of the cases. Various factors were identified as contributors to postoperative hypocalcemia, including lymph node metastasis (odds ratio [OR] = 2.6; P < 0.05), total thyroidectomy (OR = 8.0; P < 0.01), diminished parathyroid hormone (PTH) levels (OR = 12.6; P < 0.001), and reduced 25-hydroxyvitamin D3 (25[OH]D3) levels (P < 0.01). Furthermore, multivariate analyses revealed that free thyroxine (FT4) (P = 0.04), 25(OH)D3 (P = 0.037), surgical procedure (P < 0.001), and cancer stage (P < 0.001) independently predicted postoperative hypocalcemia. Notably, our findings underscored a substantial correlation between total thyroidectomy (OR = 21.5, P < 0.001), diminished PTH levels (P < 0.001), and the occurrence of postoperative hypoparathyroidism.

**Conclusions:**

The identification of lymph node metastasis, total thyroid surgery, decreased PTH and 25(OH)D3 levels, and albumin concentration are crucial factors in guiding the surgical team to prevent the onset of hypocalcemia.

## 1. Background

The incidence of thyroid cancer has increased, becoming a global health concern (1). Thyroid surgery, including total thyroidectomy and subtotal thyroidectomy, remains the primary treatment option for patients. Despite being widely utilized, thyroidectomy poses the risk of severe complications such as unintended parathyroid removal, postoperative bleeding, hypocalcemia, and recurrent laryngeal nerve injury (2, 3). Notably, hypocalcemia emerges as the most common complication following thyroid surgery, underscoring its significance in enhancing patient care (4, 5). 

Postoperative hypocalcemia is a frequently encountered complication following thyroid surgery, adding a layer of complexity to the management of patients with thyroid cancer (5). The occurrence of postoperative transient hypocalcemia has been documented to vary between 7% and 51% (5, 6). The acute phase of postoperative hypocalcemia unfolds within the initial 24 hours following thyroid surgery, with symptoms such as numbness and tingling emerging around the oral region before progressing to the extremities (hands, fingers, feet, and toes) (7). 

The intricate relationship between thyroid surgery and hypocalcemia is underscored by its potential to occur independently, especially in cases of parathyroid damage. Notably, 5% of patients exhibited symptoms of hypocalcemia, with the possibility of persistence up to six months postoperatively. Despite the prevalence of postoperative hypocalcemia, it is crucial to recognize its transient nature, observed in approximately 60 - 70% of cases (8).

This transient phase underscores the importance of proactive measures, with studies indicating that calcium supplementation administered within the first month after surgery can effectively prevent and manage postoperative hypocalcemia. If hypocalcemia and hyperphosphatemia persist for more than a year, hypoparathyroidism is considered permanent, and the patient will require long-term treatment with oral calcium and vitamin D (7).

This background sets the stage for a comprehensive exploration of the epidemiological nuances, clinical manifestations, and management strategies associated with postoperative hypocalcemia in the context of thyroid cancer. A deeper understanding of these interconnections is imperative for healthcare professionals to enhance patient care, improve surgical outcomes, and contribute to the evolving landscape of thyroid cancer management. In Vietnam, recent studies have primarily focused on assessing the effectiveness of thyroid cancer treatment methods without exploring postoperative hypocalcemia status, as well as related factors and management strategies to address this issue. This study aimed to describe hypocalcemia after thyroidectomy and evaluate several factors associated with postoperative hypocalcemia.

## 2. Objectives

This study aimed to describe hypocalcemia following thyroidectomy and evaluate several factors associated with postoperative hypocalcemia.

## 3. Methods

### 3.1. Study Design

This cross-sectional study was conducted on patients with thyroid cancer who were scheduled for surgery at Hanoi Medical University Hospital in Hanoi, Vietnam.

### 3.2. Participants

The study included patients who were diagnosed with thyroid cancer and had undergone histopathological examination with an indication for thyroidectomy at the Oncology and Palliative Care Department of Hanoi Medical University Hospital. The data collection period was from July to December 2021. During this period, a total of 129 patients with thyroid cancer were indicated for surgery. Among them, six patients with conditions that pose a risk of hypocalcemia, such as chronic kidney disease, distal tubular acidosis syndrome, Fanconi syndrome, septicemia, and alcohol addiction, were excluded. Chronic kidney disease is defined as kidney damage or a glomerular filtration rate (GFR) of < 60 mL/min/1.73 m^2^ for 3 months or more, irrespective of the cause. Twenty-one patients who underwent lymph node dissection surgery from the second occurrence onwards (due to cancer recurrence or reoperation) and those with concurrent diagnoses of other cancers were also excluded. Additionally, 11 patients refused to participate in the study. As a result, 91 patients were included in the analysis.

#### 3.2.1. Clinical setting

The study investigated participants who met the eligibility criteria to determine their baseline characteristics, including demographic and clinical features. Before undergoing surgery, the participants' free thyroxine (FT4), free triiodothyronine (FT3), thyroid-stimulating hormone (TSH), total calcium, 25(OH)D3, and complete blood count were measured. During the surgery, the surgical approach, intraoperative complications, and postoperative diagnosis were recorded. Additionally, the participants were followed for 24 hours post-operation, during which their total calcium, ionized calcium, parathyroid hormone (PTH), albumin, and phosphorus levels were tested.

#### 3.2.2. Laboratory Measurements

The 25-hydroxyvitamin D (25[OH]D3), TSH, FT3, and FT4 levels were measured using the electrochemiluminescence immunoassay (ECLIA) on the Roche Cobas E801 Immunology analyzer. Total calcium and albumin levels were measured using the spectrophotometric method on the Roche Cobas C501 analyzer. Plasma PTH levels were measured by the electrochemiluminescence immunoassay method using a Roche Cobas E601 analyzer.

### 3.3. Sample Size and Sampling

The sample size was calculated based on the formula for estimating one proportion:


$$n=\frac{Z_{\left(1-\frac{\alpha }{2}\right)}^{2}\times \left(p\left(1-p\right)\right)}{{∆}^{2}}$$

In this study, the sample size (n) was determined based on the prevalence (p) of postoperative hypocalcemia in patients with thyroid cancer, as obtained from a previous investigation (38.6%) (7). The absolute precision (Δ) was set at 0.1, and the significance level (α) was set at 0.05. With Z_(1-α/2)_ equal to 1.96, the calculated sample size was 91 patients. A convenience sampling method was employed for participant selection.

### 3.4. Definitions Variables

This study incorporated various indicators and research variables to comprehensively analyze the impact of thyroidectomy on patients with thyroid cancer. The variables were categorized into different domains for systematic investigation:

- General information and clinical characteristics: Age, sex, anatomical diagnosis, stage of cancer, and surgical method.

- Variables related to hypocalcemia: Laboratory tests conducted within the initial 24 hours post-thyroidectomy, focusing on a diagnostic threshold of serum calcium levels less than 8.5 mg/dL (< 2.1 mmol/L).

- Variables for analyzing the relationship between hypocalcemia and clinical and subclinical characteristics: Concentrations of 25(OH)D3, PTH, TSH, FT4, blood phosphorus, and serum albumin. The concentration of 25(OH)D3 is considered decreased when the level is ≤ 30 ng/mL (8). The diagnosis of hypoparathyroidism is made when the PTH concentration is below 14 pg/mL (< 1.5 pmol/L) and the serum calcium concentration is below 8.5 mg/dL (< 2.1 mmol/L) (9).

Measurement data with a normal distribution were expressed as the mean ± standard deviation (SD) and were compared using the two-sample *t*-test. Categorical data were expressed as percentages and were compared between groups using the chi-square test or Fisher’s exact test. Logistic regression analysis was also performed to estimate the odds ratios (ORs) for certain parameters. Statistical significance was set at P < 0.05. The Pearson correlation coefficient was used to explore the correlation between two quantitative variables. Data analysis was performed using Stata version 18.0 (College Station, TX: StataCorp LLC).

### 3.5. Ethical Consideration

Informed consent was obtained from all participants involved in the study, emphasizing the voluntary nature of their participation, the purpose of the research, and the confidentiality of their information. The study was conducted in accordance with the Declaration of Helsinki and other relevant ethical guidelines, prioritizing the well-being and rights of participants throughout the research process.

## 4. Results

### 4.1. Characteristics of Participants

A total of 91 patients with thyroid cancer were included in this study. The participants’ mean age was 43.65 ± 13.3 years. Among them, 45% were under 40 years old and 38.5% were in the age range of 40 - 59 years. Females constituted most of the sample (83.5%). The majority of patients had stage I (90.1%) and papillary thyroid carcinoma (PTC) (91.2%). Nearly half of the patients who underwent thyroidectomy experienced lymph node metastasis, accounting for 47.2%. The mean levels of total calcium pre- and postoperatively were 2.31 ± 0.08 mmol/L and 2.14 ± 0.12 mmol/L, respectively. The mean level of postoperative PTH was 2.34 ± 1.11 pmol/L. Postoperative hypocalcemia and hypoparathyroidism occurred in 25 (27.5%) and 14 (15.4%) patients, respectively (Table 1). 

**Table 1.** Baseline Characteristics of Enrolled Patients ^a^

Variables	Total of Patients (N = 91)		
	Total (n = 91)	Male (n = 15)	Female (n = 76)
**Age (y)**	43.65 ± 13.3		
18 - 39	41 (45.0)	8 (53.3)	33 (43.4)
40 - 59	35 (38.5)	3 (20.0)	32 (42.1)
≥ 60	15 (16.5)	4 (26.7)	11 (14.5)
**BMI (kg/m** ^**2**^ **)**			
< 18.5	6 (6.6)	0 (0.0)	6 (7.9)
18.5 - 22.9	59 (64.8)	6 (40.0)	53 (69.7)
≥ 23	26 (28.6)	9 (60.0)	17 (22.4)
**Stage of cancer**			
Stage I	82 (90.1)	13 (86.7)	69 (90.8)
Stage II	9 (9.9)	2 (13.3)	7 (9.2)
**Pathology**			
PTC	83 (91.2)	14 (93.3)	69 (90.8)
Others	8 (8.8)	1 (6.7)	7 (9.2)
**Lymph node metastasis**			
No	48 (52.8)	6 (40.0)	42 (55.3)
Yes	43 (47.2)	9 (60.0)	34 (44.7)
**Surgical procedure**			
Lobectomy	49 (53.8)	7 (46.7)	42 (55.3)
Total thyroidectomy	42 (46.2)	8 (53.3)	34 (44.7)
**Preoperative total calcium (mmol/L)**	2.31 ± 0.08	2.35 ± 0.078	2.307 ± 0.08
**Postoperative total calcium (mmol/L)**	2.14 ± 0.12	2.16 ± 0.16	2.14 ± 0.11
**Postoperative PTH (pmol/L)**	2.34 ± 1.11	2.47 ± 0.9	2.31 ± 1.14
**Preoperative 25(OH)D3 (ng/mL)**	23.1 ± 11.0	26.2 ± 14.2	22.5 ± 10.3
**Postoperative hypocalcemia**			
No	66 (72.5)	12 (80.0)	54 (71.1)
Yes	25 (27.5)	3 (20.0)	22 (28.9)
**Postoperative hypoparathyroidism**			
No	77 (84.6)	14 (93.3)	63 (82.9)
Yes	14 (15.4)	1 (6.7)	13 (17.1)

Abbreviations: BMI, body mass index; PTC, papillary thyroid carcinoma; SD, standard deviation.

^a^ Values are expressed as mean ± SD unless otherwise indicated.

### 4.2. Postoperative Hypocalcemia and Hypoparathyroidism

**Figure 1.**
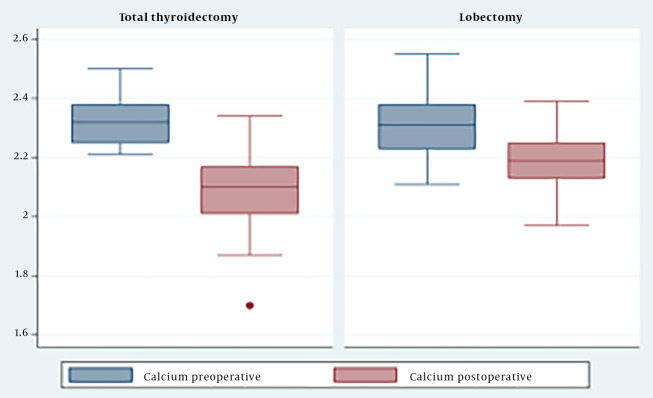
Change in calcium levels by surgical method

The results indicated that the serum calcium concentration after surgery was significantly lower than before surgery (P < 0.05) (Figure 1). Specifically, in the group that underwent total thyroidectomy, the serum calcium concentration decreased from 2.3 ± 0.07 mmol/L to 2.08 ± 0.1 mmol/L. In the group that underwent thyroid lobectomy, the serum calcium concentration decreased from 2.3 ± 0.1 mmol/L to 2.19 ± 0.87 mmol/L (Figure 1). 

**Figure 2.**
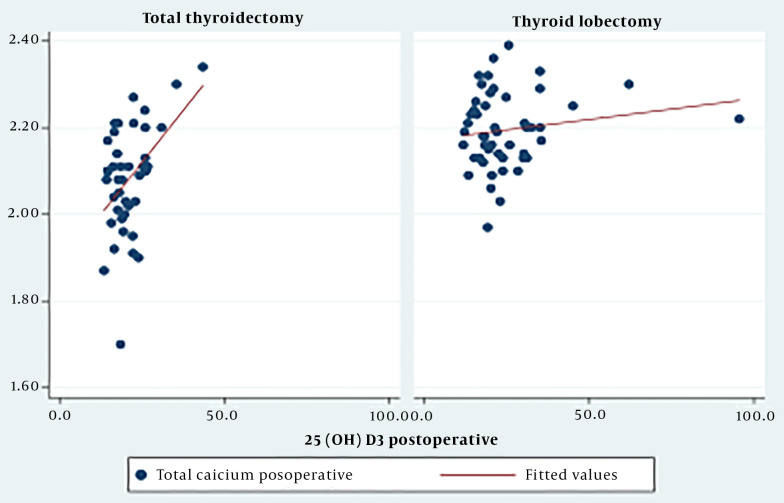
Postoperative correlation between 25(OH)D3 and serum calcium by surgical method

The study found a moderate positive correlation (r = 0.46, P < 0.05) between the 25(OH)D3 concentration and postoperative serum calcium levels in the total thyroidectomy group (Figure 2). 

Table 2 presents a comparison of the clinical and subclinical characteristics of the two groups. Four factors were found to be significantly associated with postoperative hypocalcemia: Lymph node metastasis (P = 0.049, OR = 2.6), total thyroidectomy (P = 0.001, OR = 8.0), 25(OH)D3 level (P = 0.0005), and PTH level (P < 0.001, OR = 12.6). However, sex, age group, cancer stage, pathology, and levels of FT3 and FT4 were not significantly correlated with postoperative hypocalcemia (P > 0.05). Additionally, the study identified potential risk factors for postoperative hypoparathyroidism, revealing that the surgical procedure (P = 0.0002) and PTH level (P < 0.001) were significantly associated with postoperative hypoparathyroidism (Table 2). 

**Table 2.** Relationship Between Hypocalcemia and Clinical Characteristics of Patients (N = 91)

Variables	Postoperative Hypocalcemia				Postoperative Hypoparathyroidism			
	No (n = 66)	Yes (n = 25)	OR (95%CI)	P-Value	No (n = 77)	Yes (n = 14)	OR (95%CI)	P-Value
**Gender**				0.4				0.3
Male	12 (80.0)	3 (20.0)	1		14 (93.3)	1 (6.7)	1	
Female	54 (71.1)	22 (29.0)	1.6 (0.4 - 6.4)		63 (82.9)	13 (17.1)	2.9 (0.3 - 24.5)	
**Age groups (y)**				0.3				0.48
< 40	30 (73.2)	11 (26.8)	1		35 (85.4)	6 (14.6)	1	
40 - 59	23 (65.7)	12 (34.3)	1.4(0.5 - 3.8)		28 (80.0)	7 (20.0)	1.46 (0.4 - 4.9)	
≥ 60	13 (86.7)	2 (13.3)	0.4 (0.1 - 2.2)		14 (93.3)	1 (6.7)	0.4 (0.04 - 3.9)	
**Cancer stage**				0.5				0.6
Stage I	59 (72.0)	23 (28.1)	1		69 (84.2)	13 (15.8)	1	
Stage II	7 (77.8)	2 (22.2)	0.7 (0.1 - 3.8)		8 (88.9)	1 (11.1)	0.7 (0.1 - 5.8)	
**Pathology**				0.3			-	0.25
PTC	59 (71.1)	24 (28.9)	1		69 (83.1)	14 (16.9)		
Others	7 (87.5)	1 (12.5)	0.4 (0.04 - 3.1)		8 (100.0)	0 (0.0)		
**Lymph node metastasis**				0.049				0.05
No	39 (81.3)	9 (18.8)	1		44 (91.7)	4 (8.3)	1	
Yes	27 (62.8)	16 (37.2)	2.6 (1 - 6.8)		33 (76.7)	10 (23.3)	3.3 (0.93 - 12)	
**Surgical procedure**				0.001				0.0002
Lobectomy	44 (89.8)	5 (10.2)	1		48 (97.9)	1 (2.1)	1	
Total thyroidectomy	22 (52.4)	20 (47.6)	8.0 (2.4 - 27.2)		29 (69.1)	13 (30.9)	21.5 (2.1 - 211.9)	
**FT3**				0.9				0.5
≥ 3.1 pmol/L	63 (72.4)	24 (27.6)	1		74 (85.1)	13 (14.9)	1	
< 3.1 pmol/L	3 (75.0)	1 (25.0)	0.88 (0.09 - 8.9)		3 (75.0)	1 (25.0)	1.9 (0.2 - 20.0)	
**FT4**				0.7				0.76
≥ 11.5 pmol/L	50 (73.5)	18 (26.5)	1		58 (85.3)	10 (14.7)	1	
< 11.5 pmol/L	16 (68.6)	7 (30.4)	1.2 (0.4 - 3.5)		19 (82.6)	4 (17.4)	1.2 (0.3 - 4.4)	
**25(OH)D3**				0.005				0.06
> 30 ng/mL	15 (100.0)	0 (0.0)	-		15 (100)	0 (0.0)	-	
≤ 30 ng/mL	51 (67.1)	25 (32.9)			62 (81.6)	14 (18.4)		
**PTH**				< 0.0001				< 0.0001
≥ 1.5 pmol/L	59 (85.5)	10 (14.5)	1		69 (100)	0 (0.0)	-	
< 1.5 pmol/L	7 (31.8)	15 (68.2)	12.6 (3.4 - 47.0)		8 (36.4)	14 (63.6)		

Abbreviations: PTH, parathyroid hormone; PTC, papillary thyroid carcinoma; OR, odd ratio; CI, confidence interval.

### 4.3. Association Factors for Postoperative Hypocalcemia

Multivariate analysis showed that the independent predictors of postoperative hypocalcemia were FT4 (P = 0.04), 25(OH)D3 (P = 0.037), surgical procedure (P < 0.001), and cancer stage (P < 0.001) (Table 3). 

**Table 3.** Multivariable Regression for Association Factors for Postoperative Hypocalcemia

Variables	Coef.	P-Value	95%CI
**PTH (pmol/L)**	0.017	0.17	- 0.008; 0.04
**FT4 (pmol/L)**	0.007	0.04	0.0002; 0.015
**25(OH)D3 (ng/mL)**	0.002	0.037	0.0001; 0.004
**Phosphorus (mmol/L)**	0.06	0.14	- 0.02; 0.14
**Albumin (g/L)**	0.003	0.18	- 0.0019; 0.009
**Surgical procedure (lobectomy vs total thyroidectomy)**	0.11	0.000	0.05; 0.17
**Stage of cancer (stage I vs stage II)**	0.08	0.027	0.01; 0.15

Abbreviations: PTH, parathyroid hormone; FT4, free thyroxine; CI, confidence interval.

## 5. Discussion

Hypocalcemia and hypoparathyroidism are common complications associated with thyroid surgery. These conditions typically occur in the initial days after surgery and may or may not present with clinical symptoms. The mechanism of postoperative hypocalcemia after thyroidectomy is multifactorial, involving factors such as surgical technique, the surgeon's skill level, adjacent thyroid gland injury (trauma, hematoma, infarction, local ischemia), the extent of thyroid gland removal, parathyroid gland status, malignancy, sex, the presence of thyroiditis, and postoperative serum calcium reduction (9, 10).

In our investigation, a notable prevalence of postoperative hypocalcemia was observed, particularly within the cohort that underwent total thyroidectomy, comprising nearly fifty percent of the cases. Consequently, the preemptive identification and mitigation of predisposing risk factors and prognostic indicators for postoperative hypocalcemia hold promise in furnishing clinicians with enhanced therapeutic modalities and surveillance protocols, thereby improving the overall standard of patient care. The recorded spectrum of postoperative transient hypocalcemia incidence ranges from 7% to 51% in previous studies (5, 6, 11). This fluctuation in the reported rate stems from the various reasons mentioned above. 

Additionally, when the vascular pedicle sustains an injury, compromising all glands through resection or unintended surgical manipulation, there is a sudden and substantial decrease in PTH levels, resulting in a more pronounced and rapid onset of hypocalcemia, consequently manifesting symptoms (4). Furthermore, our investigation revealed a statistically significant reduction in serum calcium concentration after surgery compared to pre-surgery levels. 

Del Rio et al.’s research demonstrated an average postoperative decrease in calcium content of 1.203 ± 0.41 mg/dL (approximately 0.3 mmol/L) (12). Multiple studies have indicated that a decline in serum calcium concentration within the initial 24 hours post-surgery, compared to pre-surgery levels, is associated with transient hypocalcemia (13-15). Del Rio et al.’s findings further highlighted a significant disparity in serum calcium levels between the preoperative and immediate postoperative periods in the transient hypocalcemia group (1.67 ± 0.49 mg/dL), which was statistically significant when compared to the group without hypocalcemia (0.92 ± 0.47 mg/dL), with a p-value less than 0.001 (12).

In terms of the factors associated with hypocalcemia, this study revealed a correlation with distinct surgical approaches. The total thyroidectomy group had a higher hypocalcemia rate. Similarly, Del Rio et al.’s 2019 study demonstrated a statistically significant difference in postoperative calcium concentration between the total thyroidectomy and thyroid lobectomy groups (38.8% vs. 13.8%, P < 0.001) (12). Prolonged surgical stimulation increases the release of calcitonin due to the large surgical scope of malignant thyroid disease and the need for lymph node dissection, leading to a decrease in blood calcium (12). If the scope of the surgery is extensive and the surgical field is unclear, it is easy to mishandle the parathyroid glands (16). The removal of lymph and adipose tissue may cause avascular necrosis of the parathyroid glands, which may result in hypocalcemia in patients (17). Hence, the choice of surgical method should be tailored to the patient's specific disease type to prevent an unwarranted broadening of the surgical scope.

Furthermore, we found a significant association between lymph node metastasis and hypocalcemia. The group with lymph node dissection had a 37.2% higher rate of hypocalcemia than the group without lymph node dissection (18.8%); this difference was statistically significant (P < 0.05, OR = 2.6).

Hypoparathyroidism has been confirmed as an independent risk factor for hypocalcemia after thyroid surgery because parathyroid gland function directly affects serum calcium levels (18, 19). This result was consistent with previous studies and the known pathogenesis of the parathyroid gland. The mechanisms underlying PTH reduction are related to the disruption of the thyroid artery supply or venous drainage, mechanical trauma, or accidental partial resection of the parathyroid gland. Normal parathyroid gland function requires an abundant blood supply (20). Parathyroid blood supply is both delicate and complex and requires close attention during thyroidectomy to ensure preservation of the parathyroid gland.

A study by Rubin et al. in 2020 on 517 patients with thyroid cancer also showed that the hypocalcemia group had a lower PTH concentration (14.8 ± 15.9 pg/mL) than the group without hypocalcemia (40.2 ± 28.5 pg/mL) with P < 0.01 (21). In addition, PTH measurements in published studies ranged from 10 minutes to 24 hours after thyroidectomy. Studies have shown that a low recovery rate predicts postoperative PTH levels (< 12 pg/mL) with 100% sensitivity and 92% specificity for the development of hypocalcemia (22). Additionally, low levels of postoperative PTH four hours after surgery (3 - 10 pg/mL) have a sensitivity of 90% and specificity of 84% for predicting postoperative hypocalcemia (23). Thus, the earliest opportunity to predict hypocalcemia is to measure blood PTH levels as soon as possible after thyroid surgery. A PTH level < 15 pg/mL is usually a predictor of impending hypocalcemia. The measurement of PTH alone during surgery or in combination with serum calcium measurement may guide the decision to discharge or follow up in the hospital, prescribe oral calcium supplementation, or take a more aggressive approach to prevent and treat hypocalcemia (20).

Our study also showed that the 25(OH)D3 concentration in the hypocalcemia group was lower than in the group without hypocalcemia (P < 0.05). This result was similar to that of a study by Rubin et al. on patients with thyroid cancer. Rubin et al. measured the concentration of 25(OH)D3 42 days before surgery, one day after surgery, and serum calcium levels adjusted for albumin. They found that the mean 25(OH)D3 concentration in the hypocalcemia group was significantly lower (P < 0.05) (21). Recently, Danan and Shonka Jr (24) demonstrated that vitamin D level is a significant predictor of postoperative hypocalcemia in patients in whom more than three parathyroid glands were identified. Additionally, preoperative PTH and vitamin D levels, along with postoperative changes in calcium levels, were biochemical predictors of hypocalcemia after thyroidectomy (21, 24, 25). Therefore, it is advisable to monitor vitamin D levels and administer vitamin D supplements to prevent the onset of hypocalcemia after surgery.

This study had some limitations. First, the study involved a relatively small sample size from one hospital. Future studies should consider designs with larger sample sizes and multiple centers. Second, the timeframe of this study was limited to in-hospital treatment. A longer follow-up period should be considered in future studies to investigate the longer-term consequences of hypocalcemia following thyroidectomy.

### 5.1. Conclusions

The identification of lymph node metastasis, total thyroid surgery, decreased PTH and 25(OH)D3 levels, and albumin concentration proved to be instrumental in guiding the surgical team toward preventing the onset of hypocalcemia. These critical findings are of great significance in ensuring safe and successful surgery, and the surgical team relies on them to make informed decisions.

## Data Availability

The dataset presented in the study is available on request from the corresponding author during submission or after publication.
